# Supplementary material for: Modeling glioblastoma heterogeneity as a dynamic network of cell states
Source: Mol Syst Biol. 2021 Sep 16;17(9):e10105. doi: 10.15252/msb.202010105 (PMC8444284; doi:10.15252/msb.202010105)
Supplement: Supplementary file 6 — Source Data for Figure 5 [file MSB-17-e10105-s004.zip › Figure5A_sourcedata/GSEA_3071/hallmarks_stateVI.GseaPreranked.1623742557031/HALLMARK_WNT_BETA_CATENIN_SIGNALING.html]

Details for gene set HALLMARK\_WNT\_BETA\_CATENIN\_SIGNALING[GSEA]

|  || Dataset | state63071 |
| Phenotype | NoPhenotypeAvailable |
| Upregulated in class | na\_pos |
| GeneSet | HALLMARK\_WNT\_BETA\_CATENIN\_SIGNALING |
| Enrichment Score (ES) | 0.30891263 |
| Normalized Enrichment Score (NES) | 1.2583276 |
| Nominal p-value | 0.19424461 |
| FDR q-value | 0.19620505 |
| FWER p-Value | 0.785 |
Table: GSEA Results Summary

  

Fig 1: Enrichment plot: HALLMARK\_WNT\_BETA\_CATENIN\_SIGNALING      
 Profile of the Running ES Score & Positions of GeneSet Members on the Rank Ordered List

  

| PROBE | GENE SYMBOL | GENE\_TITLE | RANK IN GENE LIST | RANK METRIC SCORE | RUNNING ES | CORE ENRICHMENT || 1 | DKK1 |  |  | 9 | 1.134 | 0.1479 | Yes |
| 2 | MYC |  |  | 69 | 0.815 | 0.2397 | Yes |
| 3 | WNT5B |  |  | 143 | 0.673 | 0.3089 | Yes |
| 4 | JAG1 |  |  | 876 | 0.365 | 0.1552 | No |
| 5 | CCND2 |  |  | 1064 | 0.335 | 0.1481 | No |
| 6 | JAG2 |  |  | 1281 | 0.306 | 0.1290 | No |
| 7 | PSEN2 |  |  | 1442 | 0.286 | 0.1227 | No |
| 8 | ADAM17 |  |  | 1855 | -0.258 | 0.0432 | No |
| 9 | HEY2 |  |  | 2101 | -0.297 | 0.0149 | No |
| 10 | NCOR2 |  |  | 2134 | -0.303 | 0.0462 | No |
| 11 | NCSTN |  |  | 2142 | -0.304 | 0.0846 | No |
| 12 | NKD1 |  |  | 2180 | -0.311 | 0.1156 | No |
| 13 | FZD8 |  |  | 2592 | -0.399 | 0.0551 | No |
| 14 | CTNNB1 |  |  | 2863 | -0.483 | 0.0445 | No |
| 15 | LEF1 |  |  | 3149 | -0.616 | 0.0476 | No |
| 16 | NOTCH1 |  |  | 3192 | -0.657 | 0.1231 | No |
Table: GSEA details [plain text format]

  

Fig 2: HALLMARK\_WNT\_BETA\_CATENIN\_SIGNALING: Random ES distribution      
 Gene set null distribution of ES for **HALLMARK\_WNT\_BETA\_CATENIN\_SIGNALING**
